# Peptide-Guided TiO_2_/Graphene Oxide–Cellulose Hybrid Aerogels for Visible-Light Photocatalytic Degradation of Organic Pollutants

**DOI:** 10.3390/ma18194565

**Published:** 2025-09-30

**Authors:** Haonan Dai, Wenliang Zhang, Wensheng Lei, Yan Wang, Gang Wei

**Affiliations:** 1 College of Chemistry and Chemical Engineering, Qingdao University, Qingdao 266071, China; dhn0308@outlook.com (H.D.); 15806820287@163.com (W.L.); 2 Key Laboratory of Molecular Medicine and Biotherapy, The Ministry of Industry and Information Technology, Beijing Institute of Technology, School of Life Science, Beijing 100081, China; zhangwenliang99@hotmail.com; 3 School of Polymer Science and Engineering, Qingdao University of Science and Technology, Qingdao 266042, China

**Keywords:** peptide self-assembly, titanium dioxide, biomineralization, photocatalytic degradation, hybrid aerogels

## Abstract

Titanium dioxide (TiO_2_), owing to its excellent photocatalytic performance and environmental friendliness, holds great potential in the remediation of water pollution. In this study, we introduce a green and facile strategy to fabricate TiO_2_-based hybrid aerogels, in which the peptide FQFQFIFK first self-assembles into peptide nanofibers (PNFs), followed by in situ biomineralization of TiO_2_ on the PNFs. The TiO_2_-loaded PNFs are then combined with graphene oxide (GO) via π–π interactions and integrated with microcrystalline cellulose (MCC) to construct a stable three-dimensional (3D) porous framework. The resulting GO/MCC/PNFs-TiO_2_ aerogels exhibit high porosity, low density, and good mechanical stability. Photocatalytic experiments show that the aerogels efficiently degrade various organic dyes (methylene blue, rhodamine B, crystal violet, and Orange II) and antibiotics (e.g., tetracycline) under visible-light irradiation, achieving final degradation efficiencies higher than 90%. The excellent performance is attributed to the synergistic effect of the ordered interface provided by the PNF template, the stabilization and uniform dispersion facilitated by GO, and the mechanically robust 3D scaffold constructed by MCC. This work provides an efficient and sustainable strategy for designing functional hybrid aerogels and lays a foundation for their application in water treatment and environmental remediation.

## 1. Introduction

With the rapid development of industrialization and urbanization, large quantities of dyes, antibiotics, and other persistent organic pollutants are continuously discharged into aquatic environments, posing severe threats to ecosystems and public health [1,2,3]. Conventional physical or chemical removal methods often suffer from limited efficiency, high energy consumption, or secondary pollution. Therefore, the development of sustainable, renewable, and highly stable photocatalytic materials has become a key strategy for environmental remediation [4,5,6,7,8]. Photocatalysis, which directly utilizes solar energy to degrade pollutants, has attracted widespread attention. Among photocatalysts, titanium dioxide (TiO_2_), owing to its tunable band gap, chemical stability, and environmental friendliness, has attracted wide attention in both photocatalytic and optoelectronic applications [9,10,11,12,13]. By tuning the structure and composition of TiO_2_ nanomaterials, Belkhanchi et al. prepared sol–gel-derived thin films incorporating nitrogen-doped carbon nanotubes (N-CNTs), which were shown to significantly influence their optical properties, charge carrier dynamics, and photoactivity [14]. In recent years, TiO_2_ has achieved remarkable progress in organic dye degradation. For example, Yang et al. designed cobalt-doped rutile TiO_2_ nanorods that exhibited highly efficient adsorption and photocatalytic activity toward methylene blue (MB) under visible light [15]. Similarly, Qutub et al. found that TiO_2_ nanoparticles demonstrated excellent photocatalytic activity in the degradation of Acid Blue dye, mainly attributed to their large surface area and enhanced bandgap [16]. Nevertheless, TiO_2_ still faces challenges such as a wide bandgap [17], low visible-light utilization [18], and rapid recombination of photogenerated electron–hole pairs [19]. To overcome these limitations, strategies such as elemental modification [20], heterostructure construction [21], and creation of carbon-based composites [22] have been developed to enhance visible-light activity and charge separation efficiency [23]. Among these approaches, biomineralization has emerged as a promising biomimetic strategy owing to its mild conditions, low energy consumption, and precise structural regulation [24,25]. Based on the molecular self-assembly of peptides or proteins, this method can effectively guide the ordered nucleation and growth of inorganic nanoparticles, enabling refined control over structure and the construction of environmentally friendly composite systems with tight interfacial integration [26,27]. For instance, Zhang et al. successfully fabricated Ti_3_C_2_T_x_–TiO_2_ composites using wool keratin peptides as linking media, achieving significantly enhanced photocatalytic activity for dye and antibiotic degradation, which was attributed to peptide-mediated interfacial contact and charge transfer [28]. Furthermore, biomimetic sol–gel strategies have been employed to synthesize TiO_2_ and other inorganic oxides, enabling controlled nucleation and morphology adjustment under mild conditions by mimicking molecular regulation in natural mineralization processes [29].

In addition, the incorporation of carbon-based materials has been recognized as one of the most effective strategies to enhance photocatalytic performance of TiO_2_ [30]. Among them, graphene oxide (GO), with its high specific surface area, abundant oxygen-containing functional groups, and excellent electrical conductivity, has emerged as a promising candidate for constructing high-performance photocatalytic composites [31,32]. The two-dimensional (2D) structure of GO provides abundant π–π conjugation sites and surface defects, which not only facilitate uniform TiO_2_ nanoparticle loading but also significantly suppress electron–hole recombination through interfacial charge transfer [33,34]. Recent studies have demonstrated that GO/TiO_2_ heterostructures exhibited superior light absorption and pollutant degradation performance. For instance, Jain et al. reported that TiO_2_–graphene nanocomposites showed enhanced photocatalytic activity for MB degradation [35]. In such heterojunctions, oxygen vacancies in TiO_2_ can trap photogenerated electrons, while GO modulates the bandgap and promotes electron transport, facilitating directional electron migration from TiO_2_ to GO and thereby improving photocatalytic efficiency [36]. Wang et al. fabricated one-dimensional GO/TiO_2_ photonic crystals via sol–gel and spin-coating methods, which effectively enhanced tetracycline degradation efficiency [37]. From a macroscopic perspective, cellulose-based materials, particularly microcrystalline cellulose (MCC) and nanocellulose, have been widely utilized as renewable supports owing to their high mechanical strength and abundant hydroxyl groups, making them ideal scaffolds for photocatalytic aerogels [38,39]. The MCC framework not only provides a stable three-dimensional (3D) network but also generates hierarchical pores during freeze-drying, thereby enhancing pollutant adsorption and interfacial reactions [40]. For example, Amaly et al. employed MCC as a flexible support for photocatalysts to construct porous MCC aerogels, improving both mechanical stability and surface area, which significantly enhanced tetracycline degradation efficiency [41]. The synergistic effect of MCC and GO not only provided a stable 3D framework but also facilitated the uniform dispersion and strong interfacial binding of TiO_2_ nanoparticles, thereby promoting effective utilization of active sites and rapid charge migration.

In this study, a biomineralization strategy was employed to synthesize TiO_2_ for photocatalytic degradation of organic dyes and antibiotics. The peptide sequence FQFQFIFK was selected for peptide self-assembly to form peptide nanofibers (PNFs) as biotemplates for TiO_2_ mineralization. The octapeptide FQFQFIFK was chosen for its balanced amino acid composition, enabling rapid self-assembly into nanofibers that provide binding sites for Ti^4+^ precursors and GO sheets, promote in situ TiO_2_ nucleation, and allow biomineralization under mild conditions, avoiding high-temperature calcination that could damage the MCC scaffold. Subsequently, TiO_2_ nanoparticles were in situ deposited onto PNFs using titanium(IV) bis(ammonium lactate)dihydroxide (TBALDH) as a precursor. TiO_2_-loaded PNFs were further integrated with GO through π–π interactions, followed by noncovalent assembly with MCC to form a hybrid framework. Finally, freeze-drying produced 3D hybrid aerogels with a highly porous architecture. The resulting GO/MCC/PNFs-TiO_2_ aerogels exhibited high porosity, low density, good mechanical stability, and excellent photocatalytic performance in degrading multiple dyes and antibiotics under visible light.

## 2. Materials and Methods

### 2.1. Materials

All chemicals used in this study were of analytical grade. The peptide with the sequence FQFQFIFK was purchased from SynPeptide Biotechnology Co., Ltd. (Nanjing, China). Monolayer GO aqueous dispersions (GO-1, 10 mg/g) were obtained from Hangzhou Gaoxi Technology Co., Ltd. (Hangzhou, China). Titanium(IV) bis(ammonium lactate)dihydroxide (TBALDH, 50 wt% in water) was obtained from Ron Reagents. Ascorbic acid (AA), ammonium oxalate (AO), and isopropanol (IPA), microcrystalline cellulose (MCC), methylene blue (MB), rhodamine B (RhB), Orange II (AO7), crystal violet (CV), and tetracycline (TC) were purchased from Shanghai Maclean Biochemical Technology Co., Ltd. (Shanghai, China).

### 2.2. Self-Assembly of Peptides and Biomimetic Mineralization of TiO_2_

A total of 10 mg of FQFQFIFK peptide monomers was dissolved in 20 mL of deionized water (DW) and ultrasonicated for 5 min to ensure uniform dispersion, yielding a 0.5 mg/mL peptide solution. The vials were incubated in a water bath at 37 °C, and samples were collected every 12 h for up to 36 h. After 36 h of incubation, 100 μL of TBALDH solution was added to the peptide solution and reacted at 37 °C for 24 h. The resulting suspension was drop-cast onto freshly cleaved mica sheets for characterization. Subsequently, the suspension was cooled to room temperature and centrifuged at 10,000 rpm to remove the supernatant. The precipitate was washed three times with DW, yielding a pale-yellow powder for further use.

### 2.3. Preparation of GO/MCC/PNFs-TiO_2_ Hybrid Aerogels

**Scheme 1:**
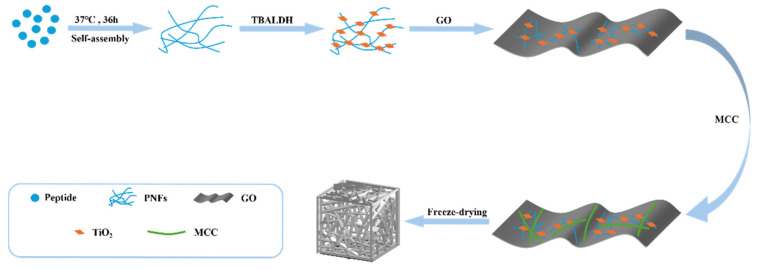
Schematic presentation of the synthesis of GO/MCC/PNFs-TiO_2_ hybrid aerogels.

GO/MCC/PNFs-TiO_2_ hybrid aerogels were prepared via a biomineralization, adsorption, and freeze-drying process (Scheme 1). First, 1 g of a 1 wt% GO dispersion was mixed with 10 mL of deionized water and stirred at 300 rpm for 2 h to obtain a 1 mg/mL GO suspension. The pale-yellow PNFs–TiO_2_ precipitate was then added to the GO suspension and stirred for 12 h, allowing PNFs–TiO_2_ complexes to be uniformly anchored on the GO nanosheets. Subsequently, 40 mg of MCC was ultrasonicated for 30 min and added to the GO/PNFs–TiO_2_ suspension, followed by stirring for an additional 12 h. The mixture was transferred into plastic molds and frozen at −20 °C for 12 h. Finally, freeze-drying was performed at –80 °C under a vacuum of 20 Pa for 24 h, yielding porous GO/MCC/PNFs-TiO_2_ aerogels. The mechanical strength and porosity of the hybrid aerogels could be tuned by varying the MCC-to-GO ratio.

### 2.4. Photocatalytic Tests

The photocatalytic activity of the as-synthesized GO/MCC/PNFs-TiO_2_ hybrid aerogel was evaluated by monitoring the degradation of tetracycline under visible-light irradiation. The experiments were conducted in a customized photoreactor illuminated by a white LED light source. The emission spectrum of the LED featured a primary peak at 449 nm and a broad Stokes-shifted band between 500 and 600 nm (Figure S1). The light intensity (PM100D, THORLABS) was maintained constant at 200 mW/cm^2^ for all experiments to accurately assess and compare the photocatalytic performance.

Briefly, 20 mg of GO/MCC/PNFs-TiO_2_ hybrid aerogel was added to 100 mL of aqueous solutions of MB, CV, RhB, AO7, or TC (20 mg/L). The experiments were conducted in a double-jacketed beaker, with circulating coolant in the outer jacket to maintain the reaction temperature. The mixed solutions were first stirred in the dark for 60 min to achieve adsorption–desorption equilibrium, followed by irradiation with visible light. At 30 min intervals, 3 mL aliquots were withdrawn, filtered through a 0.22 μm membrane, and the absorbance was measured to calculate the removal efficiency. Absorbance measurements were taken at 664, 590, 554, 483, and 357 nm for MB, CV, RhB, AO7, and TC, respectively. To validate the reproducibility of the experimental results, triplicate parallel samples were analyzed under identical conditions. The data are shown with error bars reflecting the standard deviation across replicates.

### 2.5. Characterization Techniques

Samples for atomic force microscopy (AFM) were prepared by depositing 10 μL of the peptide or PNFs–TiO_2_ suspension onto freshly cleaved mica sheets and drying at room temperature for 24 h. AFM imaging was performed in tapping mode using an FM-Nanoview 6800 (FSM-Precision, Suzhou, China) equipped with a Tap300Al-G silicon probe (300 kHz, 40 N/m). The images were processed using Gwyddion software (version 2.57). The morphology of GO/PNFs–TiO_2_ suspensions was characterized by transmission electron microscopy (TEM, HT7700, Hitachi, Tokyo, Japan). The porous structures of GO/MCC/PNFs-TiO_2_ aerogels were examined using scanning electron microscopy (SEM, JSM-6390LV, JEOL, Tokyo, Japan). The crystalline structure of TiO_2_ was analyzed by X-ray diffraction (XRD, SmartLab 3 kW, Rigaku, Tokyo, Japan). X-ray photoelectron spectroscopy (XPS) was performed using a PHI 5000 VersaProbe III spectrometer (ULVAC-PHI, Chigasaki, Japan). UV–Vis diffuse reflectance spectra (UV–Vis DRS) and absorption properties were recorded with a spectrophotometer (T9, Beijing Puxi General Instrument Co., Ltd., Beijing, China). Electrochemical impedance spectra (EIS) were obtained from an electrochemical workstation (CHI660E, Chenhua Instrument Co., Ltd., Shenzhen, China).

## 3. Results and Discussion

### 3.1. Self-Assembly and Characterizations of PNFs–TiO_2_ Composites

**Figure 1:**
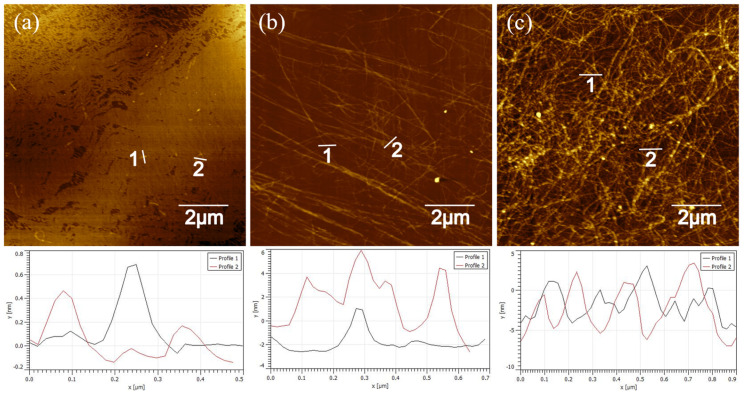
AFM images and cross-section profiles of PNFs at different incubation times: (**a**) 12 h, (**b**) 24 h, and (**c**) 36 h.

For AFM sample preparation, 10 μL of the peptide solution was deposited onto freshly cleaved mica and dried prior to imaging; the same procedure was applied to the PNFs–TiO_2_ samples. The peptide sequence FQFQFIFK, which features alternating aromatic and hydrophobic residues, self-assembles in water primarily through hydrophobic interactions, π–π stacking, and hydrogen bonding, forming PNFs with β-sheet backbones. As shown in Figure 1a, after 12 h of incubation, most peptides remained as monomers deposited on the mica, with only a few short fibrous structures (height ~0–1 nm), indicating the initial stage of fibrillation. After 24 h, the majority of peptides assembled into slender nanofibers with heights of ~1–4 nm and lengths of 2–4 μm (Figure 1b). At 36 h, abundant nanofibers with evident stacking and interlacing appeared, exhibiting typical heights of approximately 5 nm (Figure 1c). These results confirm that FQFQFIFK forms stable, well-defined PNFs that maintain their morphology at room temperature, providing an ideal molecular framework for subsequent TiO_2_ incorporation.

**Figure 2:**
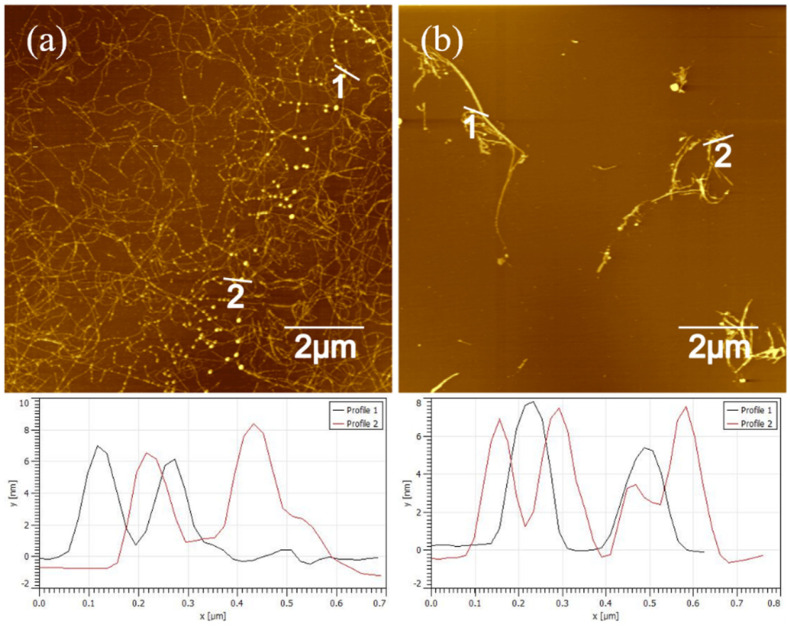
Structural features and cross-section profiles of PNFs–TiO_2_ at different concentrations: (**a**) 0.1 mg/mL and (**b**) 0.05 mg/mL.

Figure 2 illustrates the structural evolution of the PNFs following reaction with TBALDH. In Figure 2a, small nanoparticles are observed on the fiber surfaces, with local height increases of approximately 5–10 nm compared to bare PNFs. At lower precursor concentration (Figure 2b), discrete nanoparticles clearly decorate isolated fibers, exhibiting a height difference of ~6 nm, indicating partial TiO_2_ growth on the PNFs [42]. In aqueous media, TBALDH hydrolyzes to Ti–OH species, which initially bind electrostatically to lysine residues; progressive hydrogen bonding between backbone carbonyls and Ti–OH promotes nucleation on the fiber surfaces, attracting additional Ti species. Subsequent condensation of Ti–OH generates a Ti–O–Ti network, resulting in amorphous TiO_2_ anchored on the PNFs. An AFM-based TiO_2_ particle-size distribution (Figure S2) shows a unimodal, near-Gaussian distribution with an average diameter of ~95 nm (SD~20 nm, median~91 nm, *n* ≥ 100).

### 3.2. Synthesis and Characterizations of GO/PNFs–TiO_2_ Suspensions

**Figure 3:**
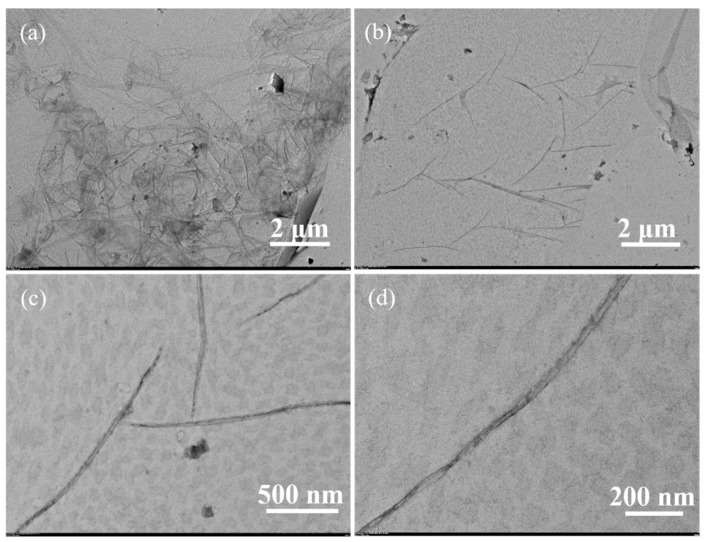
TEM images of GO/PNFs–TiO_2_ suspensions at different magnifications: (**a**,**b**) low magnification showing the fiber network; (**c**) high magnification showing multiple fibers loaded with TiO_2_; (**d**) high magnification of a single fiber with TiO_2_.

GO/PNFs–TiO_2_ suspensions were then formed through cooperative interactions between PNFs and GO. Lysine residues and backbone carbonyl groups in FQFQFIFK can form hydrogen bonds with carboxyl and hydroxyl groups on the GO surface, while phenylalanine residues engage in π–π interactions with the GO basal plane, thereby stabilizing PNFs–TiO_2_ on the 2D sheets. After phosphotungstic acid staining, TEM images (Figure 3a,b) reveal that PNFs aggregate and anchor onto the GO surface, with dark TiO_2_ nanoparticles observed around the fibers, indicating specific interactions between GO and PNFs–TiO_2_. At higher magnifications (Figure 3c), the GO surface appears flat, and individual PNFs are fully anchored without stacking. Additionally, PNFs on GO may exhibit helical morphologies decorated with TiO_2_ nanoparticles (Figure 3d). These observations confirm the successful synthesis of the GO/PNFs–TiO_2_ hybrid. The electrochemical impedance spectra of PNFs–TiO_2_ and PNFs–TiO_2_/GO (Figure S3) show relatively small semicircle diameters, indicating low charge-transfer resistance and high charge separation efficiency, which contribute to the enhanced photocatalytic performance.

### 3.3. Preparation and Characterization of GO/MCC/PNFs-TiO_2_ Hybrid Aerogels

**Figure 4:**
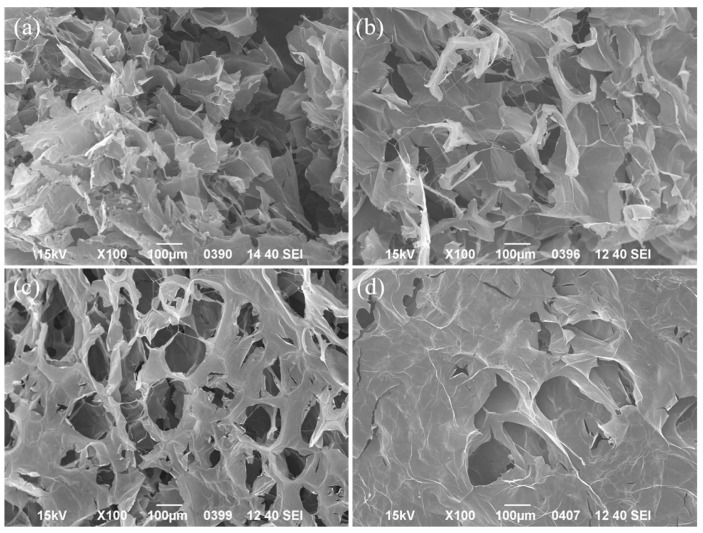
SEM images of aerogels at different MCC:GO/PNFs–TiO_2_ mass ratios: (**a**) 1:1, (**b**) 1:2, (**c**) 1:4, and (**d**) 2:1.

Although GO/PNFs–TiO_2_ suspensions exhibit photocatalytic activity, their mechanical strength is insufficient for long-term operation without a supportive matrix. Therefore, microcrystalline cellulose (MCC), a low-cost, highly crystalline, mechanically robust material rich in hydroxyl groups, was introduced, binding noncovalently with GO to form a stable 3D network. MCC not only enhances structural stability but also generates well-defined pores during freeze-drying, improving both mechanical strength and wastewater treatment performance, thereby providing an economical and reliable scaffold. As shown in Figure 4, at an MCC:GO ratio of 1:1, the interior is fragmented with poorly formed pores (Figure 4a); at 1:2, MCC binds closely with GO, but the pores remain insufficiently robust (Figure 4b); at 1:4, abundant and well-formed pores are observed, with good mechanical strength (Figure 4c); and at 2:1, excess MCC aggregates GO, yielding high strength but limited porosity (Figure 4d). Based on these results, an MCC:GO/PNFs–TiO_2_ ratio of 1:4 was selected for subsequent experiments.

**Figure 5:**
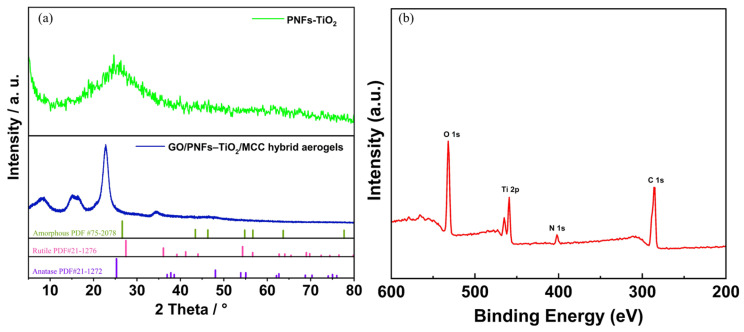
XRD pattern (**a**) and XPS survey (**b**) of GO/MCC/PNFs-TiO_2_ hybrid aerogels.

MCC-based aerogels are not heat-resistant, and high-temperature calcination can damage their porous network. To preserve the aerogel structure, the amorphous TiO_2_ produced via biomineralization was retained for subsequent experiments. In the XRD pattern (Figure 5a), the PNFs–TiO_2_ pattern exhibits a broad peak near 25° with a slight shift from anatase (101), indicating short-range order but an overall amorphous structure [43,44]. The hybrid aerogel shows a similar profile, confirming that GO and MCC do not induce TiO_2_ crystallization. Minor sharp peaks originate from GO stacking or cellulose diffraction [45]. To preserve the MCC scaffold, no high-temperature calcination was applied, intentionally retaining amorphous TiO_2_. The XPS survey (Figure 5b) reveals a clear Ti 2p signal at 458 eV, confirming the successful mineralization of Ti species on the PNFs [46].

**Figure 6:**
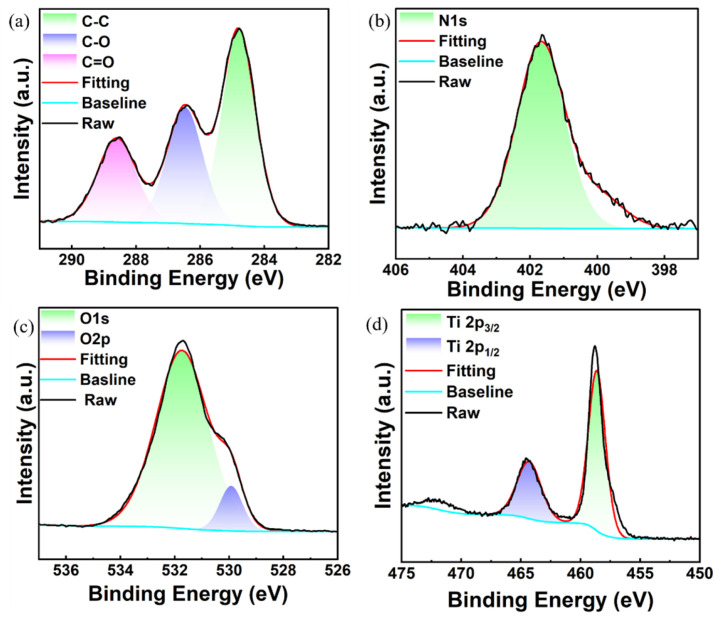
XPS core-level spectra of C 1s (**a**), N 1s (**b**), O 1s (**c**), and Ti 2p (**d**) in GO/MCC/PNFs-TiO_2_ hybrid aerogels.

High-resolution XPS further reveals pronounced C, N, O, and Ti signals. The C 1s peak at 284.8 eV corresponds to C–C/C=C bonds from the carbon frameworks of GO and MCC (Figure 6a) [47]. The N 1s peak at 401.5 eV indicates the presence of peptides on the aerogel surface (Figure 6b) [48]. The O 1s peak at 531.8 eV (–OH) suggests hydrogen bonding or coordination with peptide carbonyls/esters, facilitating the immobilization of TiO_2_ nanoparticles (Figure 6c) [49]. The Ti 2p XPS spectrum of the GO/PNFs–TiO_2_ hybrid (Figure 6d) shows peaks at 458 eV (Ti 2p_3/2_) [50,51] and 464 eV (Ti 2p_1/2_) [52,53], indicating the presence of Ti^4+^. This suggests that TiO_2_ nanoparticles nucleate uniformly and remain stable under peptide guidance, preventing aggregation and maintaining the dispersity and photocatalytic performance of the aerogel. Overall, the peptide functions as a template for TiO_2_ growth and enhances compositional stability, providing an ordered interface favorable for high photocatalytic activity.

### 3.4. Photocatalytic Degradation of Organic Pollutants

**Figure 7:**
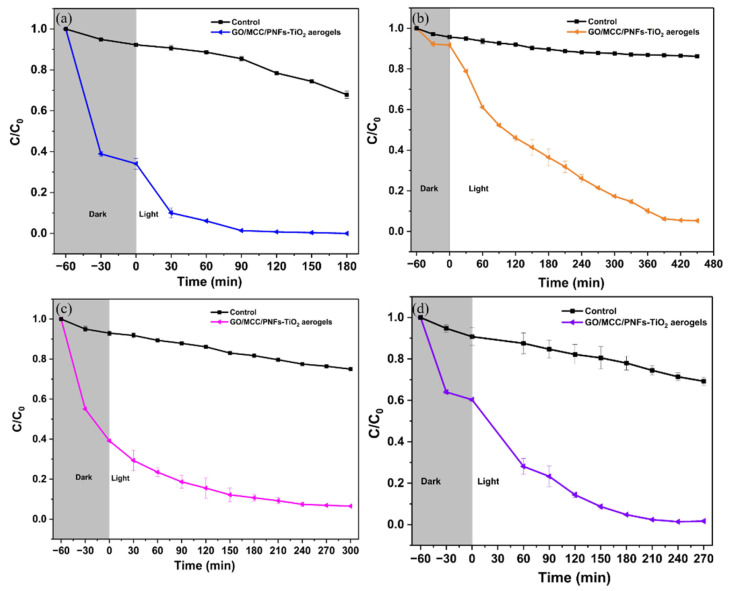
Variations in UV–Vis absorption spectra intensity during photocatalytic degradation of dye wastewaters by GO/MCC/PNFs-TiO_2_ hybrid aerogels: (**a**) MB, (**b**) AO7, (**c**) RhB, and (**d**) CV.

To evaluate the photocatalytic performance of the GO/MCC/PNFs-TiO_2_ hybrid aerogels, four model pollutants, i.e., MB, AO7, RhB, and CV, were selected. Under continuous circulation of cooling water, the reaction temperature was maintained at 20–30 °C. A total of 10 mg of aerogel was introduced into the simulated wastewater with a pollutant concentration of 20 mg/L. Owing to the highly porous structure of the aerogel and its large specific surface area, it exhibited strong adsorption capacity. TOC analysis (Table S1) showed negligible increase in dissolved organic carbon after 4 h usage, confirming the structural integrity of the hybrid aerogel and low risk of secondary contamination during the photocatalytic process. Therefore, prior to visible-light irradiation, the samples were allowed to adsorb the pollutants in the dark for 60 min to achieve adsorption–desorption equilibrium (Figure 7). Subsequently, photocatalytic degradation was performed under visible-light irradiation with an intensity of 200 mW/cm^2^. The photocatalytic efficiency of the hybrid aerogels was calculated according to Equation (1).

(1) $$\eta =(C_{0}-C)/C_{0}$$

where *η* represents the degradation rate, *C*_0_ is the initial pollutant concentration, and *C* denotes the concentration at a given time *t*. The photocatalytic degradation kinetics of five model pollutants (methylene blue, rhodamine B, crystal violet, Orange II, and tetracycline) were analyzed (Figures S4 and S5). A pseudo-first-order kinetic model was employed,

(2) $$Ln(C_{0}/C)=kt$$

where *k* is the apparent rate constant of degradation.

Figure 7a shows the photocatalytic degradation of MB. Under dark conditions, the hybrid aerogel reached adsorption equilibrium after 60 min, with an adsorption efficiency of approximately 60%. Upon subsequent illumination for 30 min, the MB concentration in solution significantly decreased to around 10%, and by 90 min, the photocatalytic degradation efficiency approached 100%. The experiment was continued up to 180 min to ensure that MB adsorbed within the aerogel was fully degraded. In comparison, the photocatalytic degradation of AO7 was more gradual: under dark conditions, adsorption equilibrium was achieved after approximately 60 min, with a removal rate of about 10%. Upon exposure to light, its concentration gradually decreased, reaching nearly complete degradation at approximately 420 min (Figure 7b). As shown in Figure 7c, RhB exhibited a removal rate of approximately 60% after 60 min of dark adsorption, indicating the material’s strong adsorption capacity toward RhB. Under subsequent light irradiation, the system reached degradation equilibrium at around 270 min, with an overall degradation rate exceeding 90%. Figure 7d illustrates that the adsorption of CV on the hybrid aerogel reached approximately 40% within 60 min, followed by a significant decrease in CV concentration during the first hour of illumination, with a photocatalytic degradation rate approaching 25%, ultimately achieving nearly 100% at 210 min. Collectively, these results demonstrate that the GO/PNFs-TiO_2_/MCC hybrid aerogel exhibits a synergistic effect of rapid adsorption and efficient photocatalytic degradation toward various organic dyes, highlighting its excellent potential for wastewater treatment applications.

**Figure 8:**
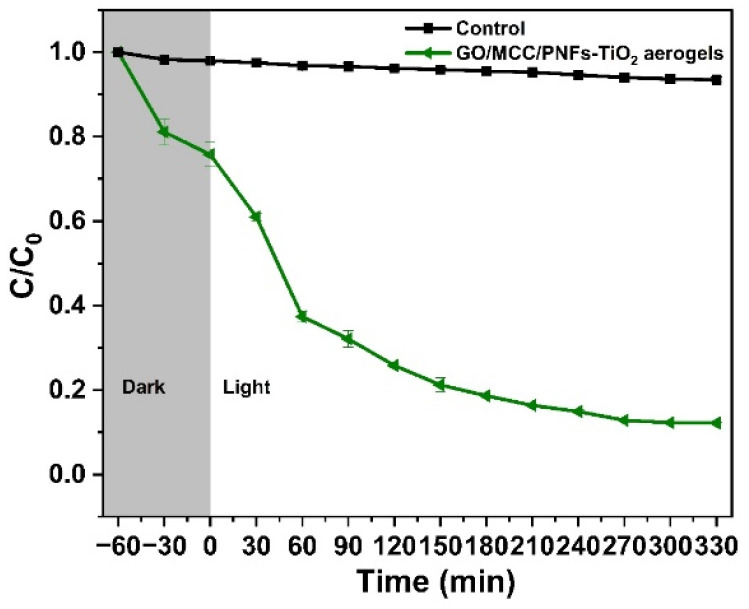
Variations in UV–Vis absorption intensity during photocatalytic degradation of TC by GO/MCC/PNFs-TiO_2_ hybrid aerogels.

Antibiotics discharged from pharmaceutical and chemical industries have emerged as significant aquatic pollutants, posing threats to ecosystems and potentially human health [36]. For TC (Figure 8), adsorption reached equilibrium after 60 min in the dark, with an adsorption efficiency of approximately 20%. Under visible-light irradiation, the TC concentration decreased significantly and stabilized after approximately 300 min, achieving a final degradation efficiency of around 90%. These results indicate that the hybrid aerogels exhibit excellent photocatalytic activity for antibiotic removal.

### 3.5. Cycling Performance and Photocatalytic Mechanism

**Figure 9:**
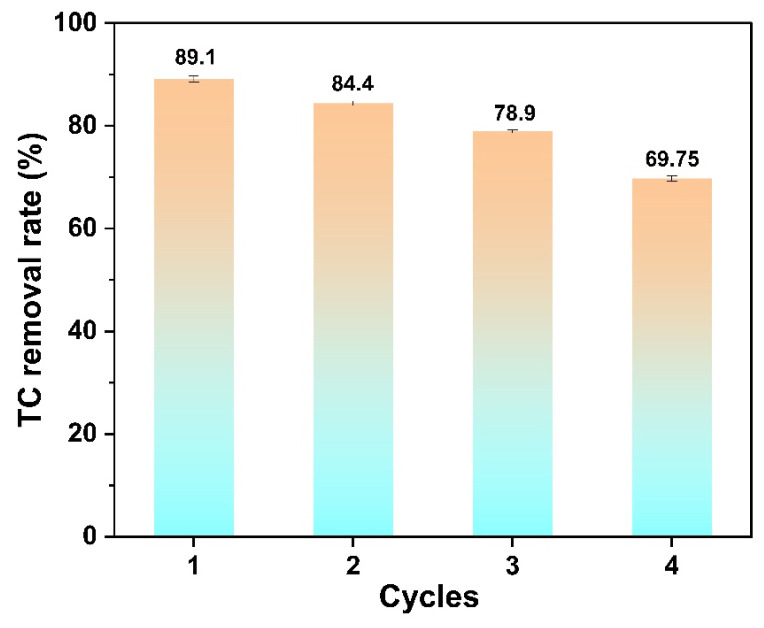
Reusability of GO/MCC/PNFs-TiO_2_ hybrid aerogels with consecutive runs.

The reusability of the GO/PNFs–TiO_2_/MCC hybrid aerogel was evaluated through four successive cycles of tetracycline degradation under visible light irradiation (Figure 9). The degradation efficiencies were 89.1%, 84.4%, 78.9%, and 69.75% for the first to fourth cycles, respectively, indicating a gradual but noticeable decline in photocatalytic activity. This decrease is likely due to the partial loss of active sites, slight TiO_2_ aggregation, and/or surface fouling by residual organic molecules. These results demonstrate that the hybrid aerogel possesses reasonable recyclability, though further regeneration strategies may be necessary to enhance activity retention for long-term applications.

**Figure 10:**
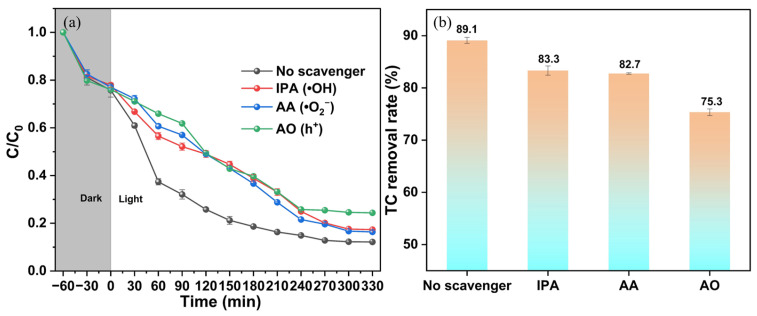
The effects of different scavengers on TC degradation profiles (**a**) and removal efficiencies (**b**) for the GO/MCC/PNFs-TiO_2_ hybrid aerogel.

To further clarify the photocatalytic mechanism, quenching experiments were performed using ascorbic acid (AA), ammonium oxalate (AO), and isopropanol (IPA) as scavengers for •O_2_^−^, h^+^, and •OH,0 respectively. As shown in Figure 10, the photocatalytic activity was significantly suppressed by all scavengers, confirming the involvement of these reactive species in the degradation process. Among them, AO caused the most pronounced inhibition, lowering the degradation efficiency to approximately 75% of the control, which indicates that photogenerated holes (h^+^) dominate the reaction pathway. The notable re-duction observed with AA and IPA also demonstrates that •O_2_^−^ and •OH play synergistic roles. Overall, these results suggest that the photocatalytic mechanism of the hybrid aero-gel is primarily h^+^-driven, with additional contributions from •O_2_^−^ and •OH radicals.

## 4. Conclusions

In summary, we presented a green and facile strategy for the fabrication of GO/MCC/PNFs-TiO_2_ hybrid aerogels. In this approach, peptides first underwent self-assembly, followed by TiO_2_ biomineralization, after which the peptide material formed a composite with GO and MCC through π–π interactions. Photocatalytic experiments demonstrated that the aerogel exhibits strong adsorption capacity under dark conditions and achieves degradation rates exceeding 90% for typical organic dyes (MB, AO7, RhB, CV) under visible-light irradiation, while also showing excellent photocatalytic removal efficiency for the antibiotic TC. These results confirm that even when TiO_2_ remains in an amorphous state, it can exhibit remarkable photocatalytic performance through the synergistic effects of multiple components. The superior performance of the proposed aerogels is primarily attributed to the interfacial effects of the peptide template, the excellent electron-transport capability of GO, and the stable 3D framework provided by MCC. Overall, this work not only provides a green, sustainable, and efficient strategy for developing novel functional hybrid aerogels but also establishes a solid foundation for their application in water pollution treatment and environmental remediation. It is expected that further optimization of peptide sequence design and precise control over TiO_2_ distribution will enable more efficient and tunable photocatalytic performance, thereby promoting the broader application of such hybrid materials in environmental management and biomedical fields.

## Data Availability

The original contributions presented in this study are included in the article/Supplementary Materials. Further inquiries can be directed to the corresponding authors.

## Supplementary Materials

The following supporting information can be downloaded at: https://www.mdpi.com/article/10.3390/ma18194565/s1, Figure S1: Emission spectrum of white LED lamp used for irradiation; Figure S2: Statistical analysis of the average particle size of TiO_2_; Figure S3: EIS plots of PNFs-TiO_2_ and PNFs-TiO_2_/GO; Figure S4: Pseudo first order kinetics of (a) MB (20 mg/L),(b) AO7 (20 mg/L),(c) RhB (20 mg/L) and (d) CV (20 mg/L) degradation; Figure S5: Pseudo first order kinetics of TC (20 mg/L) degradation; Table S1: TOC analysis and mass loss percentage of hybrid aerogel after immersion test.
