# Supplementary material for: Peptide-Guided TiO2/Graphene Oxide–Cellulose Hybrid Aerogels for Visible-Light Photocatalytic Degradation of Organic Pollutants
Source: Materials (Basel). 2025 Sep 30;18(19):4565. doi: 10.3390/ma18194565 (PMC12525821; doi:10.3390/ma18194565)
Supplement: Supplementary file 1 [file materials-18-04565-s001.zip › materials-3884995-supplementary.pdf]

Supplementary Information:

Peptide-Guided TiO_2/Graphene Oxide–Cellulose Hybrid Aerogels for Visible-Light Photocatalytic Degradation of Organic Pollutants

Haonan Dai [1], Wenliang Zhang [3], Wensheng Lei [1], Yan Wang [1,*], and Gang Wei [2,*]

[1] College of Chemistry and Chemical Engineering, Qingdao University, Qingdao 266071, P. R. China. dhn0308@outlook.com (H.D.); 15806820287@163.com (W.L.);

[2] School of Polymer Science and Engineering, Qingdao University of Science and Technology, 266042 Qingdao, China.

[3] Key Laboratory of Molecular Medicine and Biotherapy in the Ministry of Industry and Information Technology, School of Life Science, Beijing Institute of Technology, Beijing 100081, PR China zhangwenliang99@hotmail.com (W.Z.)

* Corresponding authors

E-mail address: yanwang@qdu.edu.cn (Y. W.) wei-lab@qust.edu.cn or wei@uni-bremen.de (G.W.)

Tel.: +86-15306480601 (Y.W.); +86-15066242101 (G.W.)

Table S1. TOC analysis and mass loss percentage of hybrid aerogel after immersion test

Sample	TOC (mg/L)	Net TOC Increase (mg/100 mL)	Aerogel Mass Loss (%)	Note
Blank water	1.86	/	/	Average of four blanks
Aerogel-treated water	2.145	0.0285	0.057 %	After 4 h immersion

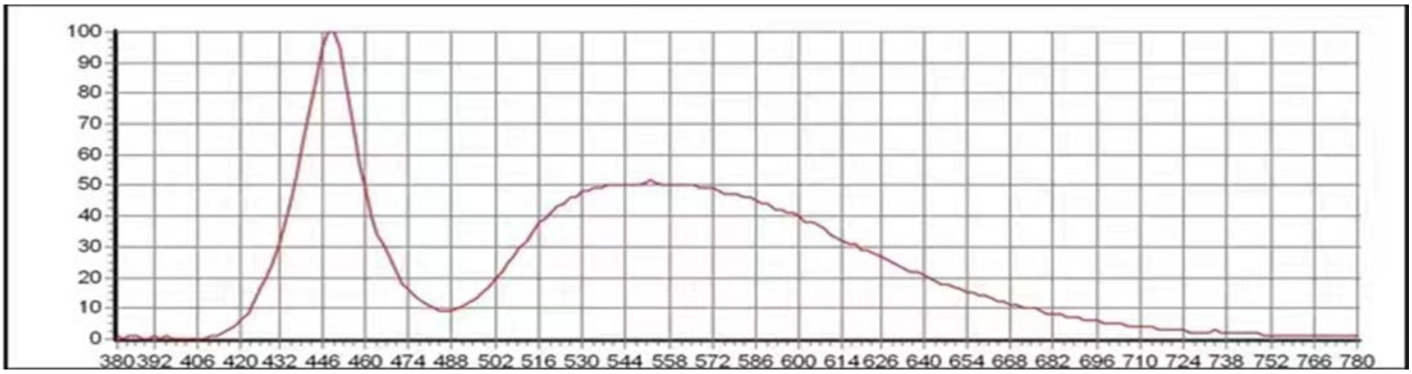

Figure S1. Emission spectrum of white LED lamp used for irradiation.

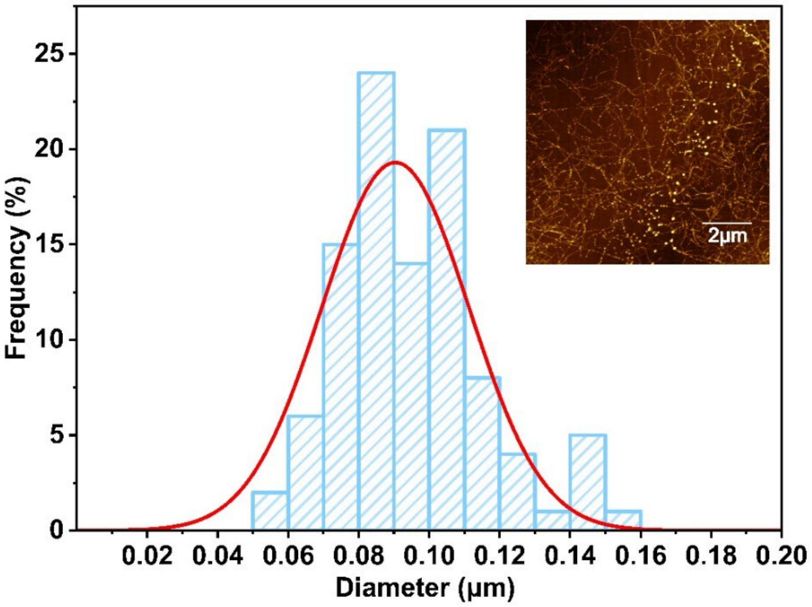

Figure S2. Statistical analysis of the average particle size of TiO_2.

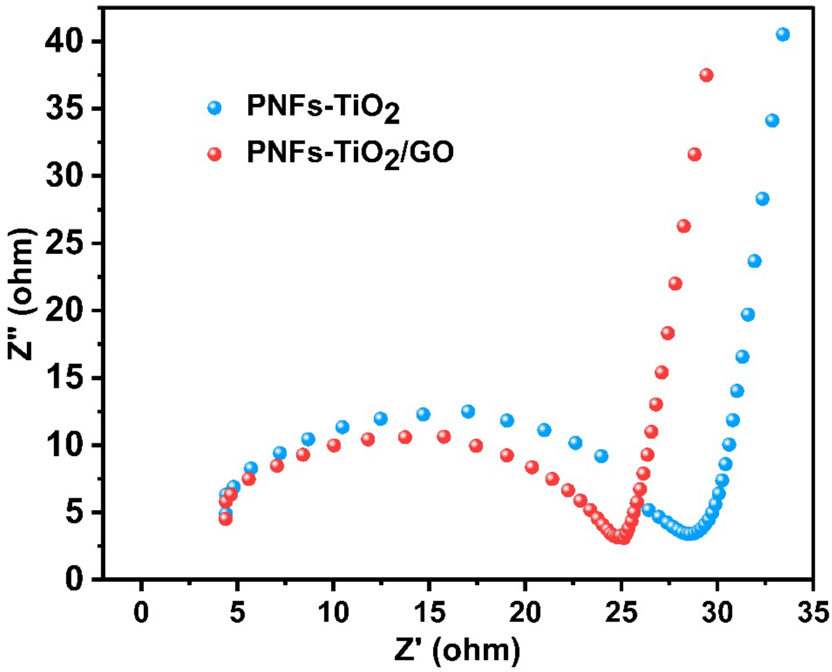

Figure S3. EIS plots of PNFs-TiO_2 and PNFs-TiO_2/GO.

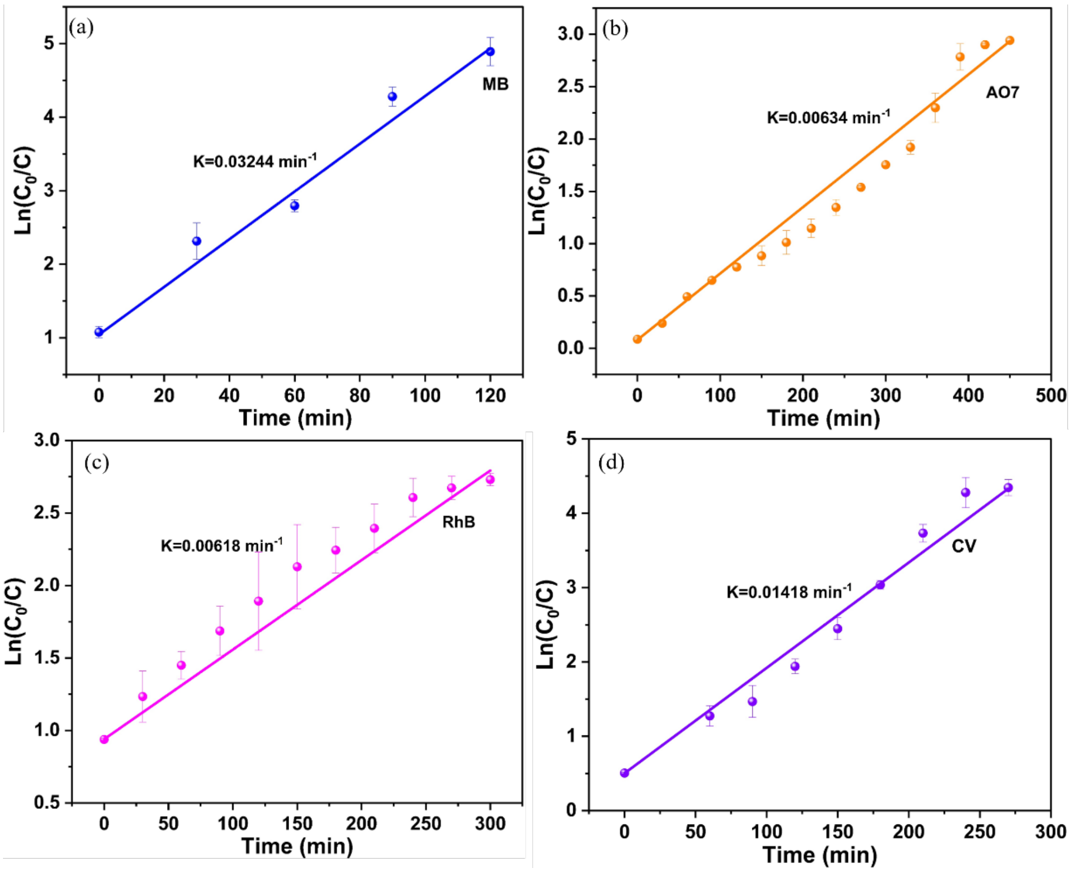

Figure S4. Pseudo first-order kinetics of (a) MB (20 mg/L),(b) AO7 (20 mg/L),(c) RhB (20 mg/L) and (d) CV (20 mg/L) degradation.

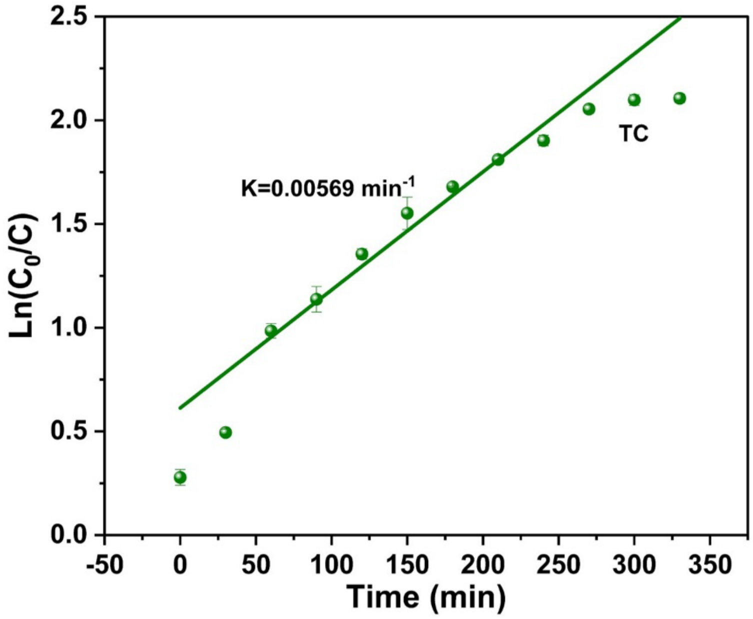

Figure S5. Pseudo first-order kinetics of TC (20 mg/L) degradation.
